# 4′-(4-Chlorophenyl)-1′-methyldispiro[indan-2,2′-pyrrolidine-3′,2′′-indan]-1,3,1′′-trione

**DOI:** 10.1107/S1600536811033642

**Published:** 2011-08-27

**Authors:** Ang Chee Wei, Mohamed Ashraf Ali, Yeong Keng Yoon, Ching Kheng Quah, Hoong-Kun Fun

**Affiliations:** a Institute for Research in Molecular Medicine, Universiti Sains Malaysia, 11800 USM, Penang, Malaysia; b X-ray Crystallography Unit, School of Physics, Universiti Sains Malaysia, 11800 USM, Penang, Malaysia

## Abstract

In the title compound, C_27_H_20_ClNO_3_, the two cyclo­pentane rings adopt envelope conformations. The pyrrolidine ring also adopts an envelope conformation (with the spiro C atom as the flap) and its least-squares plane (fitted to five atoms) makes dihedral angles of 66.50 (9), 77.36 (8) and 73.76 (8)° with the chloro­benzene ring and the two 2,3-dihydro-1*H*-indene ring systems, respectively. The mol­ecular conformation is stabilized by an intra­molecular C—H⋯O hydrogen bond, which generates an *S*(6) ring motif. In the crystal, mol­ecules are linked by C—H⋯O hydrogen bonds into chains running parallel to the [001] direction.

## Related literature

For background to the synthesis, see: Amalraj & Raghunathan (2003 ▶). For the stability of the temperature controller used for the data collection, see: Cosier & Glazer (1986 ▶). For graph-set descriptors of hydrogen-bond motifs, see: Bernstein *et al.* (1995 ▶). For related structures, see: Kumar *et al.* (2010 ▶); Wei, Ali, Choon *et al.* (2011 ▶); Wei, Ali, Ismail *et al.* (2011 ▶). For standard bond-length data, see: Allen *et al.* (1987 ▶). For ring conformations, see: Cremer & Pople (1975 ▶).
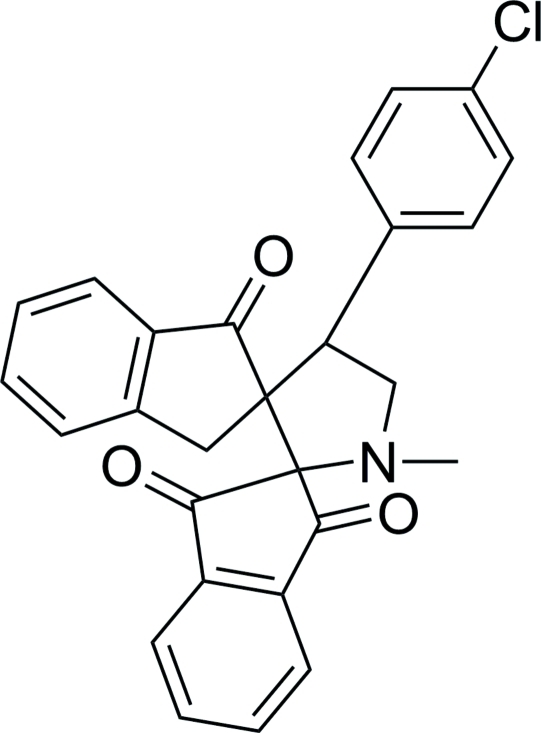


## Experimental

### 

#### Crystal data

C_27_H_20_ClNO_3_
*M* *_r_* = 441.89
Monoclinic, 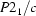

*a* = 7.8216 (1) Å
*b* = 21.2865 (3) Å
*c* = 14.0641 (2) Å
β = 116.156 (1)°
*V* = 2101.81 (5) Å^3^
*Z* = 4
Mo *K*α radiation
μ = 0.21 mm^−1^
*T* = 100 K
0.43 × 0.11 × 0.10 mm

#### Data collection

Bruker SMART APEXII CCD diffractometer
Absorption correction: multi-scan (*SADABS*; Bruker, 2009 ▶) *T*_min_ = 0.913, *T*_max_ = 0.979
25402 measured reflections
6459 independent reflections
4537 reflections with *I* > 2σ(*I*)
*R*_int_ = 0.047

#### Refinement

*R*[*F* ^2^ > 2σ(*F* ^2^)] = 0.050
*wR*(*F* ^2^) = 0.113
*S* = 1.06
6459 reflections
290 parameters
H-atom parameters constrained
Δρ_max_ = 0.31 e Å^−3^
Δρ_min_ = −0.41 e Å^−3^

### 

Data collection: *APEX2* (Bruker, 2009 ▶); cell refinement: *SAINT* (Bruker, 2009 ▶); data reduction: *SAINT*; program(s) used to solve structure: *SHELXTL* (Sheldrick, 2008 ▶); program(s) used to refine structure: *SHELXTL*; molecular graphics: *SHELXTL*; software used to prepare material for publication: *SHELXTL* and *PLATON* (Spek, 2009 ▶).

## Supplementary Material

Crystal structure: contains datablock(s) global, I. DOI: 10.1107/S1600536811033642/hb6361sup1.cif

Structure factors: contains datablock(s) I. DOI: 10.1107/S1600536811033642/hb6361Isup2.hkl

Additional supplementary materials:  crystallographic information; 3D view; checkCIF report

## Figures and Tables

**Table 1:** Hydrogen-bond geometry (Å, °)

*D*—H⋯*A*	*D*—H	H⋯*A*	*D*⋯*A*	*D*—H⋯*A*
C18—H18*A*⋯O2	0.99	2.40	3.055 (2)	123
C6—H6*A*⋯O1^i^	0.95	2.57	3.244 (2)	128
C14—H14*A*⋯O3^i^	0.95	2.45	3.167 (2)	132

Symmetry code: (i) 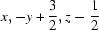.

**Fig. 1.**
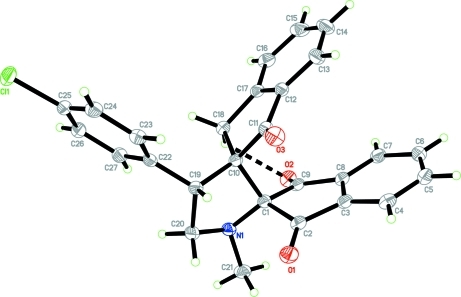
The molecular structure of the title compound showing 50% probability displacement ellipsoids for non-H atoms. The intramolecular hydrogen bond is shown as a dashed line.

**Fig. 2.**
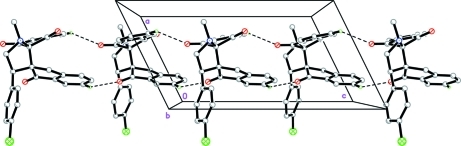
The crystal structure of the title compound, viewed along the b axis. H atoms not involved in hydrogen bonds (dashed lines) have been omitted for clarity.

## supplementary crystallographic information

### Comment

1,3-Dipolar cycloaddition is a very useful synthetic strategy to construct heterocycles in which high regio- and stereo-chemical control of peripheral substituents can be achieved (Amalraj & Raghunathan, 2003).   As part of our studies in this area, the title compound, (I), was prepared and it structure is now described.

The molecular structure is shown in Fig. 1. The two cyclopentane rings, C1-C3/C8/C9 and C10-C12/C17/C18, are in envelope conformations, puckering parameters (Cremer & Pople, 1975) Q =0.2239 (17) Å and φ = 177.0 (4)° with atom C1 at the flap; and Q = 0.2121 (17) Å and φ = 355.2 (5)° with atom C10 at the flap, respectively. Bond lengths (Allen *et al.*, 1987) and angles are within normal ranges and are comparable to related structures (Kumar *et al.*, 2010; Wei, Ali, Choon *et al.* (2011); Wei, Ali, Ismail *et al.* (2011). The pyrrolidine ring (N1/C1/C10/C19/C20) adopts an envelope conformation, puckering parameters (Cremer & Pople, 1975) Q = 0.4461 (17) Å and φ = 41.6 (2)°, with atom C1 at the flap and its least-squares plane makes dihedral angles of 66.50 (9), 77.36 (8) and 73.76 (8)° with a phenyl ring (C22-C27) and the two least-squares planes of 2,3-dihydro-1*H*-indene ring system [C1-C9 (maximum deviation of 0.234 (2) Å at atom C1) and C10-C18 (maximum deviation of 0.221 (2) Å at atom C10)], respectively. The molecular structure is stabilized by an intramolecular C18–H18A···O2 hydrogen bond (Table 1), which generates an *S*(6) ring motif (Fig. 1, Bernstein *et al.*, 1995).

In the crystal (Fig. 2), molecules are linked by C6–H6A···O1 and C14–H14A···O3 hydrogen bonds (Table 1) into one-dimensional chains parallel to [001] direction.

### Experimental

A mixture of (*E*)2-(4-chlorobenzylidene)-2,3-dihydro-*1H*- indene-1-one (0.001 mmol), ninhydrin (0.001 mmol) and sarcosine (0.002 mmol) (1:1:2) were dissolved in methanol (10 ml) and refluxed for 4 h. After completion of the reaction as evident from TLC, the mixture was poured into water (50 ml). The precipitated solid was filtered, washed with water and recrystallised from pet.ether-ethyl acetate mixture (1:1) to reveal the title compound as yellow crystals.

### Refinement

All H atoms were positioned geometrically and refined using a riding model with C–H = 0.95-1.00 Å and *U*_iso_(H) = 1.2 or 1.5 *U*_eq_(C). A rotating-group model was applied for the methyl group. The highest residual electron density peak is located at 0.73 Å from atom C10 and the deepest hole is located at 0.49 Å from atom Cl1.

### Crystal data

**Table:**

C_27_H_20_ClNO_3_	*F*(000) = 920
*M**_r_* = 441.89	*D*_x_ = 1.396 Mg m^−^^3^
Monoclinic, *P*2_1_/*c*	Mo *K*α radiation, λ = 0.71073 Å
Hall symbol: -P 2ybc	Cell parameters from 5352 reflections
*a* = 7.8216 (1) Å	θ = 2.5–30.6°
*b* = 21.2865 (3) Å	µ = 0.21 mm^−^^1^
*c* = 14.0641 (2) Å	*T* = 100 K
β = 116.156 (1)°	Needle, yellow
*V* = 2101.81 (5) Å^3^	0.43 × 0.11 × 0.10 mm
*Z* = 4	

### Data collection

**Table:**

Bruker SMART APEXII CCD diffractometer	6459 independent reflections
Radiation source: fine-focus sealed tube	4537 reflections with *I* > 2σ(*I*)
graphite	*R*_int_ = 0.047
φ and ω scans	θ_max_ = 30.7°, θ_min_ = 1.9°
Absorption correction: multi-scan (*SADABS*; Bruker, 2009)	*h* = −11→11
*T*_min_ = 0.913, *T*_max_ = 0.979	*k* = −28→30
25402 measured reflections	*l* = −20→19

### Refinement

**Table:**

Refinement on *F*^2^	Primary atom site location: structure-invariant direct methods
Least-squares matrix: full	Secondary atom site location: difference Fourier map
*R*[*F*^2^ > 2σ(*F*^2^)] = 0.050	Hydrogen site location: inferred from neighbouring sites
*wR*(*F*^2^) = 0.113	H-atom parameters constrained
*S* = 1.06	*w* = 1/[σ^2^(*F*_o_^2^) + (0.0402*P*)^2^ + 0.7009*P*] where *P* = (*F*_o_^2^ + 2*F*_c_^2^)/3
6459 reflections	(Δ/σ)_max_ = 0.003
290 parameters	Δρ_max_ = 0.31 e Å^−^^3^
0 restraints	Δρ_min_ = −0.41 e Å^−^^3^

### Special details

**Table:**

Experimental. The crystal was placed in the cold stream of an Oxford Cryosystems Cobra open-flow nitrogen cryostat (Cosier & Glazer, 1986) operating at 100.0 (1) K.
Geometry. All esds (except the esd in the dihedral angle between two l.s. planes) are estimated using the full covariance matrix. The cell esds are taken into account individually in the estimation of esds in distances, angles and torsion angles; correlations between esds in cell parameters are only used when they are defined by crystal symmetry. An approximate (isotropic) treatment of cell esds is used for estimating esds involving l.s. planes.
Refinement. Refinement of F^2^ against ALL reflections. The weighted R-factor wR and goodness of fit S are based on F^2^, conventional R-factors R are based on F, with F set to zero for negative F^2^. The threshold expression of F^2^ > 2sigma(F^2^) is used only for calculating R-factors(gt) etc. and is not relevant to the choice of reflections for refinement. R-factors based on F^2^ are statistically about twice as large as those based on F, and R- factors based on ALL data will be even larger.

### Fractional atomic coordinates and isotropic or equivalent isotropic displacement parameters (Å^2^)

**Table:**

	*x*	*y*	*z*	*U*_iso_*/*U*_eq_	
Cl1	1.36538 (5)	1.10023 (2)	0.43329 (4)	0.03235 (12)	
O1	0.34773 (16)	0.84249 (6)	0.29205 (9)	0.0267 (3)	
O2	0.21665 (15)	0.90315 (5)	−0.05015 (9)	0.0238 (2)	
O3	0.76083 (16)	0.81058 (5)	0.28215 (9)	0.0259 (3)	
N1	0.32278 (17)	0.95682 (6)	0.16129 (10)	0.0186 (3)	
C1	0.3793 (2)	0.89484 (7)	0.14450 (12)	0.0174 (3)	
C2	0.3456 (2)	0.83915 (7)	0.20561 (12)	0.0192 (3)	
C3	0.3038 (2)	0.78302 (7)	0.13665 (12)	0.0195 (3)	
C4	0.3012 (2)	0.71988 (8)	0.16125 (13)	0.0226 (3)	
H4A	0.3236	0.7071	0.2305	0.027*	
C5	0.2648 (2)	0.67623 (8)	0.08182 (14)	0.0248 (3)	
H5A	0.2625	0.6328	0.0970	0.030*	
C6	0.2312 (2)	0.69460 (8)	−0.02046 (13)	0.0247 (3)	
H6A	0.2087	0.6636	−0.0732	0.030*	
C7	0.2303 (2)	0.75755 (8)	−0.04577 (13)	0.0222 (3)	
H7A	0.2052	0.7703	−0.1154	0.027*	
C8	0.2672 (2)	0.80142 (7)	0.03387 (12)	0.0185 (3)	
C9	0.2784 (2)	0.87074 (7)	0.02905 (12)	0.0187 (3)	
C10	0.60072 (19)	0.90486 (7)	0.18206 (12)	0.0163 (3)	
C11	0.7080 (2)	0.84175 (7)	0.20191 (12)	0.0182 (3)	
C12	0.7325 (2)	0.82736 (7)	0.10625 (12)	0.0180 (3)	
C13	0.7860 (2)	0.77077 (8)	0.07756 (14)	0.0247 (3)	
H13A	0.8190	0.7354	0.1234	0.030*	
C14	0.7896 (2)	0.76766 (9)	−0.01978 (14)	0.0302 (4)	
H14A	0.8221	0.7293	−0.0424	0.036*	
C15	0.7456 (2)	0.82058 (9)	−0.08488 (14)	0.0300 (4)	
H15A	0.7504	0.8178	−0.1511	0.036*	
C16	0.6949 (2)	0.87731 (9)	−0.05530 (13)	0.0246 (3)	
H16A	0.6668	0.9132	−0.0998	0.029*	
C17	0.68632 (19)	0.88006 (7)	0.04162 (12)	0.0186 (3)	
C18	0.6335 (2)	0.93495 (7)	0.09163 (12)	0.0178 (3)	
H18A	0.5164	0.9558	0.0397	0.021*	
H18B	0.7379	0.9662	0.1197	0.021*	
C19	0.6508 (2)	0.94344 (7)	0.28455 (12)	0.0178 (3)	
H19A	0.6749	0.9126	0.3427	0.021*	
C20	0.4657 (2)	0.97897 (8)	0.26487 (12)	0.0215 (3)	
H20A	0.4259	0.9692	0.3210	0.026*	
H20B	0.4843	1.0249	0.2637	0.026*	
C21	0.1256 (2)	0.96362 (8)	0.14474 (13)	0.0251 (3)	
H21A	0.0396	0.9477	0.0744	0.038*	
H21B	0.0983	1.0081	0.1499	0.038*	
H21C	0.1069	0.9396	0.1989	0.038*	
C22	0.8282 (2)	0.98346 (7)	0.31889 (12)	0.0178 (3)	
C23	1.0004 (2)	0.96051 (8)	0.39638 (12)	0.0200 (3)	
H23A	1.0037	0.9202	0.4261	0.024*	
C24	1.1670 (2)	0.99542 (8)	0.43099 (12)	0.0219 (3)	
H24A	1.2835	0.9792	0.4836	0.026*	
C25	1.1605 (2)	1.05421 (8)	0.38765 (13)	0.0219 (3)	
C26	0.9925 (2)	1.07773 (8)	0.30868 (13)	0.0220 (3)	
H26A	0.9903	1.1176	0.2779	0.026*	
C27	0.8274 (2)	1.04207 (7)	0.27535 (12)	0.0208 (3)	
H27A	0.7117	1.0581	0.2217	0.025*	

### Atomic displacement parameters (Å^2^)

**Table:**

	*U*^11^	*U*^22^	*U*^33^	*U*^12^	*U*^13^	*U*^23^
Cl1	0.01834 (18)	0.0288 (2)	0.0454 (3)	−0.00521 (15)	0.00986 (18)	−0.00980 (19)
O1	0.0324 (6)	0.0327 (7)	0.0189 (6)	−0.0058 (5)	0.0150 (5)	−0.0018 (5)
O2	0.0238 (5)	0.0275 (6)	0.0181 (6)	−0.0004 (5)	0.0074 (5)	0.0036 (5)
O3	0.0294 (6)	0.0252 (6)	0.0228 (6)	0.0031 (5)	0.0111 (5)	0.0051 (5)
N1	0.0156 (5)	0.0206 (7)	0.0203 (7)	0.0007 (5)	0.0084 (5)	−0.0025 (5)
C1	0.0158 (6)	0.0189 (7)	0.0181 (8)	−0.0009 (5)	0.0081 (6)	−0.0007 (6)
C2	0.0164 (6)	0.0238 (8)	0.0179 (8)	−0.0017 (6)	0.0079 (6)	0.0005 (6)
C3	0.0167 (6)	0.0228 (8)	0.0202 (8)	−0.0028 (6)	0.0094 (6)	−0.0012 (6)
C4	0.0208 (7)	0.0269 (8)	0.0221 (8)	−0.0016 (6)	0.0114 (6)	0.0028 (7)
C5	0.0249 (8)	0.0204 (8)	0.0330 (10)	−0.0022 (6)	0.0162 (7)	0.0007 (7)
C6	0.0261 (8)	0.0244 (8)	0.0281 (9)	−0.0047 (6)	0.0159 (7)	−0.0070 (7)
C7	0.0213 (7)	0.0269 (9)	0.0204 (8)	−0.0048 (6)	0.0109 (6)	−0.0030 (7)
C8	0.0158 (6)	0.0213 (8)	0.0190 (8)	−0.0019 (5)	0.0083 (6)	0.0004 (6)
C9	0.0152 (6)	0.0239 (8)	0.0184 (8)	−0.0024 (5)	0.0088 (6)	−0.0013 (6)
C10	0.0152 (6)	0.0176 (7)	0.0170 (7)	−0.0004 (5)	0.0078 (6)	−0.0002 (6)
C11	0.0162 (6)	0.0187 (7)	0.0188 (8)	−0.0014 (5)	0.0068 (6)	0.0000 (6)
C12	0.0141 (6)	0.0207 (8)	0.0181 (8)	−0.0013 (5)	0.0061 (6)	−0.0033 (6)
C13	0.0208 (7)	0.0237 (8)	0.0283 (9)	0.0015 (6)	0.0097 (7)	−0.0051 (7)
C14	0.0238 (8)	0.0347 (10)	0.0322 (10)	0.0005 (7)	0.0123 (7)	−0.0149 (8)
C15	0.0229 (8)	0.0470 (11)	0.0225 (9)	−0.0024 (7)	0.0122 (7)	−0.0105 (8)
C16	0.0193 (7)	0.0362 (10)	0.0181 (8)	−0.0019 (6)	0.0083 (6)	−0.0012 (7)
C17	0.0138 (6)	0.0243 (8)	0.0177 (8)	−0.0032 (5)	0.0069 (6)	−0.0046 (6)
C18	0.0172 (7)	0.0182 (7)	0.0180 (8)	−0.0009 (5)	0.0077 (6)	0.0007 (6)
C19	0.0171 (6)	0.0200 (8)	0.0172 (8)	−0.0013 (5)	0.0084 (6)	−0.0018 (6)
C20	0.0179 (7)	0.0247 (8)	0.0231 (8)	−0.0012 (6)	0.0100 (6)	−0.0055 (6)
C21	0.0175 (7)	0.0318 (9)	0.0273 (9)	0.0016 (6)	0.0110 (7)	−0.0020 (7)
C22	0.0173 (6)	0.0201 (8)	0.0171 (7)	0.0006 (5)	0.0086 (6)	−0.0042 (6)
C23	0.0204 (7)	0.0221 (8)	0.0181 (8)	0.0027 (6)	0.0089 (6)	0.0000 (6)
C24	0.0157 (6)	0.0282 (9)	0.0187 (8)	0.0042 (6)	0.0048 (6)	−0.0016 (7)
C25	0.0159 (7)	0.0252 (8)	0.0245 (8)	−0.0025 (6)	0.0089 (6)	−0.0080 (7)
C26	0.0206 (7)	0.0191 (8)	0.0265 (9)	0.0005 (6)	0.0105 (7)	−0.0020 (7)
C27	0.0172 (7)	0.0225 (8)	0.0204 (8)	0.0019 (6)	0.0062 (6)	−0.0021 (6)

### Geometric parameters (Å, °)

**Table:**

Cl1—C25	1.7413 (15)	C13—H13A	0.9500
O1—C2	1.2106 (18)	C14—C15	1.396 (3)
O2—C9	1.2148 (18)	C14—H14A	0.9500
O3—C11	1.2139 (18)	C15—C16	1.391 (2)
N1—C1	1.4435 (19)	C15—H15A	0.9500
N1—C21	1.4616 (18)	C16—C17	1.395 (2)
N1—C20	1.4692 (19)	C16—H16A	0.9500
C1—C9	1.547 (2)	C17—C18	1.512 (2)
C1—C2	1.553 (2)	C18—H18A	0.9900
C1—C10	1.5869 (19)	C18—H18B	0.9900
C2—C3	1.482 (2)	C19—C22	1.515 (2)
C3—C4	1.390 (2)	C19—C20	1.546 (2)
C3—C8	1.400 (2)	C19—H19A	1.0000
C4—C5	1.383 (2)	C20—H20A	0.9900
C4—H4A	0.9500	C20—H20B	0.9900
C5—C6	1.399 (2)	C21—H21A	0.9800
C5—H5A	0.9500	C21—H21B	0.9800
C6—C7	1.386 (2)	C21—H21C	0.9800
C6—H6A	0.9500	C22—C27	1.389 (2)
C7—C8	1.388 (2)	C22—C23	1.395 (2)
C7—H7A	0.9500	C23—C24	1.389 (2)
C8—C9	1.481 (2)	C23—H23A	0.9500
C10—C11	1.543 (2)	C24—C25	1.383 (2)
C10—C18	1.543 (2)	C24—H24A	0.9500
C10—C19	1.552 (2)	C25—C26	1.388 (2)
C11—C12	1.472 (2)	C26—C27	1.389 (2)
C12—C17	1.388 (2)	C26—H26A	0.9500
C12—C13	1.392 (2)	C27—H27A	0.9500
C13—C14	1.383 (2)		
			
C1—N1—C21	116.22 (12)	C16—C15—C14	121.65 (16)
C1—N1—C20	107.55 (12)	C16—C15—H15A	119.2
C21—N1—C20	114.48 (12)	C14—C15—H15A	119.2
N1—C1—C9	115.06 (12)	C15—C16—C17	118.13 (16)
N1—C1—C2	117.92 (12)	C15—C16—H16A	120.9
C9—C1—C2	101.26 (12)	C17—C16—H16A	120.9
N1—C1—C10	100.98 (11)	C12—C17—C16	119.69 (15)
C9—C1—C10	111.78 (11)	C12—C17—C18	111.45 (13)
C2—C1—C10	110.17 (12)	C16—C17—C18	128.86 (15)
O1—C2—C3	127.30 (14)	C17—C18—C10	103.97 (12)
O1—C2—C1	125.47 (14)	C17—C18—H18A	111.0
C3—C2—C1	107.19 (12)	C10—C18—H18A	111.0
C4—C3—C8	120.59 (15)	C17—C18—H18B	111.0
C4—C3—C2	129.78 (14)	C10—C18—H18B	111.0
C8—C3—C2	109.62 (13)	H18A—C18—H18B	109.0
C5—C4—C3	117.96 (15)	C22—C19—C20	115.83 (13)
C5—C4—H4A	121.0	C22—C19—C10	114.48 (12)
C3—C4—H4A	121.0	C20—C19—C10	105.05 (12)
C4—C5—C6	121.41 (15)	C22—C19—H19A	107.0
C4—C5—H5A	119.3	C20—C19—H19A	107.0
C6—C5—H5A	119.3	C10—C19—H19A	107.0
C7—C6—C5	120.78 (15)	N1—C20—C19	105.29 (12)
C7—C6—H6A	119.6	N1—C20—H20A	110.7
C5—C6—H6A	119.6	C19—C20—H20A	110.7
C6—C7—C8	117.89 (15)	N1—C20—H20B	110.7
C6—C7—H7A	121.1	C19—C20—H20B	110.7
C8—C7—H7A	121.1	H20A—C20—H20B	108.8
C7—C8—C3	121.35 (15)	N1—C21—H21A	109.5
C7—C8—C9	128.97 (14)	N1—C21—H21B	109.5
C3—C8—C9	109.68 (13)	H21A—C21—H21B	109.5
O2—C9—C8	126.87 (14)	N1—C21—H21C	109.5
O2—C9—C1	125.93 (14)	H21A—C21—H21C	109.5
C8—C9—C1	107.18 (13)	H21B—C21—H21C	109.5
C11—C10—C18	103.63 (11)	C27—C22—C23	118.29 (14)
C11—C10—C19	113.48 (12)	C27—C22—C19	122.66 (13)
C18—C10—C19	118.72 (12)	C23—C22—C19	119.05 (14)
C11—C10—C1	111.72 (12)	C24—C23—C22	121.35 (15)
C18—C10—C1	109.58 (11)	C24—C23—H23A	119.3
C19—C10—C1	99.89 (11)	C22—C23—H23A	119.3
O3—C11—C12	127.43 (14)	C25—C24—C23	118.90 (14)
O3—C11—C10	125.39 (14)	C25—C24—H24A	120.6
C12—C11—C10	107.18 (12)	C23—C24—H24A	120.6
C17—C12—C13	122.31 (15)	C24—C25—C26	121.14 (14)
C17—C12—C11	109.22 (13)	C24—C25—Cl1	119.84 (12)
C13—C12—C11	128.44 (15)	C26—C25—Cl1	119.02 (13)
C14—C13—C12	117.91 (16)	C25—C26—C27	118.99 (15)
C14—C13—H13A	121.0	C25—C26—H26A	120.5
C12—C13—H13A	121.0	C27—C26—H26A	120.5
C13—C14—C15	120.28 (16)	C26—C27—C22	121.31 (14)
C13—C14—H14A	119.9	C26—C27—H27A	119.3
C15—C14—H14A	119.9	C22—C27—H27A	119.3
			
C21—N1—C1—C9	65.60 (16)	C1—C10—C11—O3	−82.09 (18)
C20—N1—C1—C9	−164.59 (12)	C18—C10—C11—C12	−20.04 (14)
C21—N1—C1—C2	−53.86 (18)	C19—C10—C11—C12	−150.16 (12)
C20—N1—C1—C2	75.96 (15)	C1—C10—C11—C12	97.84 (13)
C21—N1—C1—C10	−173.87 (12)	O3—C11—C12—C17	−168.37 (15)
C20—N1—C1—C10	−44.06 (14)	C10—C11—C12—C17	11.70 (15)
N1—C1—C2—O1	−30.5 (2)	O3—C11—C12—C13	13.8 (3)
C9—C1—C2—O1	−156.98 (15)	C10—C11—C12—C13	−166.11 (14)
C10—C1—C2—O1	84.59 (18)	C17—C12—C13—C14	−1.0 (2)
N1—C1—C2—C3	147.34 (13)	C11—C12—C13—C14	176.57 (14)
C9—C1—C2—C3	20.88 (14)	C12—C13—C14—C15	1.7 (2)
C10—C1—C2—C3	−97.55 (13)	C13—C14—C15—C16	−0.9 (2)
O1—C2—C3—C4	−16.6 (3)	C14—C15—C16—C17	−0.8 (2)
C1—C2—C3—C4	165.63 (15)	C13—C12—C17—C16	−0.7 (2)
O1—C2—C3—C8	164.77 (15)	C11—C12—C17—C16	−178.66 (13)
C1—C2—C3—C8	−13.04 (16)	C13—C12—C17—C18	179.99 (13)
C8—C3—C4—C5	1.1 (2)	C11—C12—C17—C18	2.02 (16)
C2—C3—C4—C5	−177.40 (14)	C15—C16—C17—C12	1.6 (2)
C3—C4—C5—C6	−0.1 (2)	C15—C16—C17—C18	−179.24 (14)
C4—C5—C6—C7	−1.1 (2)	C12—C17—C18—C10	−14.68 (15)
C5—C6—C7—C8	1.2 (2)	C16—C17—C18—C10	166.07 (14)
C6—C7—C8—C3	−0.1 (2)	C11—C10—C18—C17	20.42 (14)
C6—C7—C8—C9	178.77 (14)	C19—C10—C18—C17	147.31 (12)
C4—C3—C8—C7	−1.0 (2)	C1—C10—C18—C17	−98.94 (13)
C2—C3—C8—C7	177.77 (13)	C11—C10—C19—C22	84.37 (16)
C4—C3—C8—C9	179.86 (13)	C18—C10—C19—C22	−37.69 (18)
C2—C3—C8—C9	−1.33 (16)	C1—C10—C19—C22	−156.60 (12)
C7—C8—C9—O2	17.7 (2)	C11—C10—C19—C20	−147.44 (12)
C3—C8—C9—O2	−163.28 (14)	C18—C10—C19—C20	90.49 (14)
C7—C8—C9—C1	−163.78 (14)	C1—C10—C19—C20	−28.42 (14)
C3—C8—C9—C1	15.24 (15)	C1—N1—C20—C19	25.91 (15)
N1—C1—C9—O2	28.6 (2)	C21—N1—C20—C19	156.70 (13)
C2—C1—C9—O2	156.89 (14)	C22—C19—C20—N1	131.17 (13)
C10—C1—C9—O2	−85.85 (17)	C10—C19—C20—N1	3.81 (15)
N1—C1—C9—C8	−149.97 (12)	C20—C19—C22—C27	−40.6 (2)
C2—C1—C9—C8	−21.64 (14)	C10—C19—C22—C27	81.89 (17)
C10—C1—C9—C8	95.62 (14)	C20—C19—C22—C23	140.24 (14)
N1—C1—C10—C11	164.09 (12)	C10—C19—C22—C23	−97.25 (16)
C9—C1—C10—C11	−73.08 (15)	C27—C22—C23—C24	1.1 (2)
C2—C1—C10—C11	38.68 (16)	C19—C22—C23—C24	−179.73 (13)
N1—C1—C10—C18	−81.66 (13)	C22—C23—C24—C25	0.3 (2)
C9—C1—C10—C18	41.17 (16)	C23—C24—C25—C26	−1.8 (2)
C2—C1—C10—C18	152.93 (12)	C23—C24—C25—Cl1	177.50 (12)
N1—C1—C10—C19	43.77 (13)	C24—C25—C26—C27	1.9 (2)
C9—C1—C10—C19	166.61 (12)	Cl1—C25—C26—C27	−177.41 (12)
C2—C1—C10—C19	−81.64 (14)	C25—C26—C27—C22	−0.5 (2)
C18—C10—C11—O3	160.03 (14)	C23—C22—C27—C26	−1.0 (2)
C19—C10—C11—O3	29.9 (2)	C19—C22—C27—C26	179.86 (14)

### Hydrogen-bond geometry (Å, °)

**Table:**

*D*—H···*A*	*D*—H	H···*A*	*D*···*A*	*D*—H···*A*
C18—H18A···O2	0.99	2.40	3.055 (2)	123
C6—H6A···O1^i^	0.95	2.57	3.244 (2)	128
C14—H14A···O3^i^	0.95	2.45	3.167 (2)	132

Symmetry codes: (i) *x*, −*y*+3/2, *z*−1/2.
